# Comparison of Suture Versus Bony Fixation in Meniscal Allograft Transplantation Outcomes: A Meta-analysis

**DOI:** 10.1177/03635465251319540

**Published:** 2025-03-03

**Authors:** Rahul Kakria, James Randolph Onggo, Iswadi Damasena

**Affiliations:** † Faculty of Medicine, Nursing and Health Sciences, Monash University, Melbourne, Victoria, Australia; ‡ Wangaratta Hospital, Northeast Health, Wangaratta, Victoria, Australia; § Box Hill Hospital, Eastern Health, Melbourne, Victoria, Australia; Investigation performed at Monash University, Melbourne, Australia

**Keywords:** meniscal allograft transplant (MAT), suture fixation, soft tissue fixation, bony fixation, bone bridge

## Abstract

**Background:**

Meniscal allograft transplantation replaces damaged meniscal tissue with grafts, aiming to restore knee stability and function. The method employed in the fixation of the meniscal graft—suture or bony fixation—has sparked clinical interest and ongoing discussions.

**Purpose:**

To compare suture fixation with bony fixation of the meniscal graft, with the focus on functional and clinical outcomes.

**Study Design:**

Meta-analysis and systematic review; Level of evidence, 4.

**Methods:**

Meta-analyses were performed with a multidatabase search according to PRISMA guidelines on August 15, 2023. Data from published articles meeting inclusion criteria were extracted and analyzed with an inverse variance statistical model.

**Results:**

A total of 6 studies were included consisting of 334 patients: 184 suture fixation and 150 bony fixation. No statistical analysis could be performed for clinical outcomes given the heterogeneity of raw data, but no observable trends were observed from individual studies. Suture and bony fixation showed no statistically significant difference in the risks of infection (relative risk [RR], 1.52; 95% CI, 0.29-7.80; *P* = .62), graft failure (RR, 0.86; 95% CI, 0.19-3.78; *P* = .84), graft tear (RR, 1.14; 95% CI, 0.10-13.21; *P* = .91), minor graft extrusion (RR, 0.77; 95% CI, 0.20-2.92; *P* = .70), and major graft extrusion (RR, 1.20; 95% CI, 0.28-5.07; *P* = .81).

**Conclusion:**

There was no significant difference in clinical outcomes or complications between suture and bony fixation of meniscal grafts. However, the short- to medium-term follow-up in this meta-analysis prompts the need for studies with long-term follow-up, given that meniscal allograft transplantation longevity is of utmost importance in this patient group to restore function and potentially reduce the risk of arthritis progression.

The meniscus, a crescent-shaped fibrocartilaginous structure, is vital for long-term knee stability and health.^13^ Its enhances knee joint stability, deepens the tibial plateau, absorbs shock because of its elasticity, transmits load through the joint, and provides lubrication—all of which are imperative for long-term knee health.^14^ Although previously considered functionless vestigial remnants as late as 1975, today it is a priority to preserve and repair the meniscus after injury to prevent deleterious complications such as osteoarthritis.^7,15^ Moreover, surgical resection of the meniscus has been heavily associated with increasing the risk of osteoarthritis and reducing knee function, making it a vital structure to preserve to prevent progression of osteoarthritis.^6^

Given the poor vascularity of the meniscus, there is limited healing potential of this structure.^7^ Certain meniscal tears are less likely to heal, and this is dependent on the anatomic location of the tear and the extent of vascularity to that particular zone. Generally, there exists 3 broad zones: the red-red zone (peripheral outer third), which receives the most blood supply; the red-white zone (middle third), which receives less blood supply; and finally the white-white zone (inner third), which receives no blood supply. Poor healing may typically require surgical excision of the meniscus (complete or partial), but this often leads to loss of meniscal function, the onset of symptoms, and potential osteoarthritis.^22,23^ Although we suspect that osteoarthritis is highly likely due to meniscal deficiency, there remains controversy.

Meniscal allograft transplantation (MAT) is a procedure first performed by Milachowski et al in 1984.^16^ Given the degenerative ramifications of meniscectomy, MAT was proposed as a procedure that would restore normal contact pressures of the knee and thereby delay osteoarthritis progression.^9^ Classic indications for MAT include symptomatic meniscal deficiency and the absence of advanced degenerative changes. Individuals with advanced degenerative changes, obesity, inflammatory arthritis, previous septic arthritis, or synovial disease are generally contraindicated for this procedure.^1^ Individuals with malalignment or clinically unstable knees are also contraindicated. Consideration should be given to correcting these issues at the time of MAT or by staging the correction and the MAT procedure.

Multiple factors determine the success of the MAT procedure. Of paramount importance is an accurately matched graft size in addition to bone geometry and the level of patient activity. However, a surgical factor under the control of the operating surgeon involves the fixation technique—namely, suture or bony fixation. Suture fixation, also known as soft tissue fixation, involves the graft being sutured at the anterior and posterior horns before drawing these sutures through transtibial bone tunnels and fixing them by using bone anchors or tying them over a bone bridge. On the contrary, bony fixation involves the use of bone plugs at the anterior and posterior roots of the graft or a bone block to which the anterior and posterior roots are attached. The bony fixation technique then docks the plugs or slots the bone bloc into a prepared tibia for fixation.^18^ Generally, bone plugs are used for the medial meniscus and bone slots for the lateral meniscus. Currently, there are limited cumulative results when comparing fixation methods; therefore, limited studies exist that analyze randomized controlled trials that compare both fixation methods directly. The aim and objective of this study are to compare suture versus bony fixation of the meniscal graft in terms of clinical outcomes and complications.

## Methods

### Literature Search

This meta-analysis was performed according to the PRISMA criteria (Preferred Reporting Items for Systematic Reviews and Meta-analyses).^17^ A comprehensive multidatabase search (PubMed, OVID Medline, EMBASE) was conducted from date of database inception to August 15, 2023. The Medical Subject Headings and Boolean operators utilized for this search were as follows: (meniscal transplant or meniscal allograft transplant or meniscus transplant) AND (bony fixation OR suture OR soft tissue fixation OR bone plug OR bone OR bony OR suture only OR soft tissue OR bone slot). Identified articles and their corresponding references were reviewed and considered for inclusion according to the selection criteria.

### Selection Criteria

All articles of any study design that directly compared the clinical outcomes and complications of MATs via suture-only fixation or bony fixation were considered for inclusion. Exclusion criteria included studies not written in English or peer reviewed, studies focusing on meniscal tear repair instead of MAT, and studies not directly comparing the outcomes and complications of suture-only fixation versus bony fixation. Two independent reviewers (R.K., J.R.O.) reviewed records retrieved from the initial search and excluded irrelevant ones. Titles and abstracts of the remaining articles were then screened against the inclusion criteria. All discrepancies between the reviewers were discussed with a senior author for final decision-making.

### Data Extraction

Extracted data parameters included details on study design, publication year, patient number, basic demographics, fixation methods, functional outcomes, and complications. Functional outcomes were extracted as follows: Lysholm score, Tegner score, visual analog scale score, International Knee Documentation Committee score, Knee injury and Osteoarthritis Outcome Score (KOOS), joint space narrowing, meniscal extrusion, and patient satisfaction. Complications of interest were also extracted: graft tear, graft failure, arthrofibrosis, infection, root tear, rearthroscopy for pain, and additional adverse events such as a venous thrombotic event. “Graft failure” was defined as complete graft removal, whereas “graft tear” was defined as damage to the graft resulting in partial graft removal or graft refixation (which required reoperation). A “root tear” differed from a graft tear, as a root tear referred to damage to the attachment points of the allograft, whereas a graft tear was damage to the allograft itself. “Arthrofibrosis” included any arthroscopic arthrolysis required, any arthrofibrosis present, any arthroscopic debridement, or any manipulation under anesthesia required for joint stiffness.

### Methodology Assessment

The methodology quality of the studies was assessed with the Methodological Index for Non-randomized Studies (MINORS).^21^ MINORS uses 12 criteria to assess nonrandomized comparative studies. Each criterion was scored with a 3-point system from 0 to 2 (0, not reported; 1, inadequately reported; 2, adequately reported). The ideal score is 24 points.

### Statistical Analysis

Comparative meta-analysis was performed with odds ratio and 95% CI, primarily used as summary statistics. In this meta-analysis, fixed and random effects models were tested. The fixed effects model assumed that treatment effects in each study were identical, while the random effects model assumed that variations were present among studies. Chi-square tests were used to study heterogeneity among studies. The *I*^2^ statistic was used to estimate the percentage of total variation across studies attributed to heterogeneity rather than chance. A value >50% was regarded as substantial heterogeneity. *I*^2^ can be calculated as 100% × (*Q – df*)/*Q*, with *Q* defined as Cochrane's heterogeneity statistics. If substantial heterogeneity was present, the possible clinical and methodological reasons were explored qualitatively. This meta-analysis presents results with a random effects model to account for clinical diversity and methodological variation among studies. All *P* values were 2-sided. Review Manager (version 5.4; Nordic Cochrane Centre, Cochrane Collaboration) was used for statistical analysis.

## Results

### Literature Search

**Figure 1.**
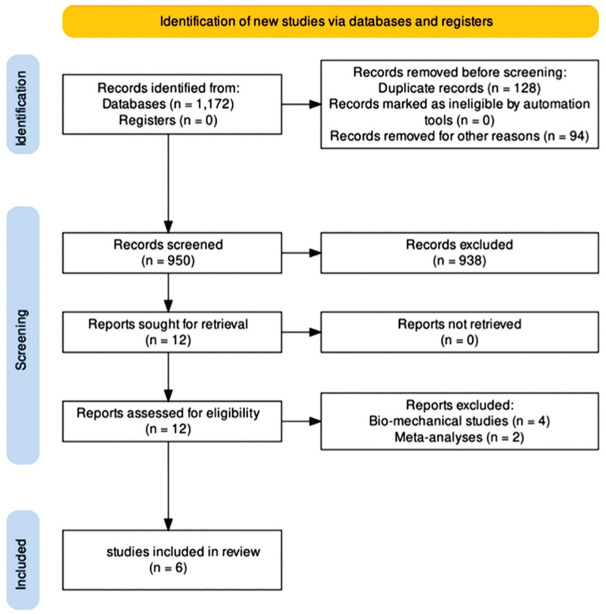
PRISMA selection process flowchart.

The PRISMA selection flowchart to identify relevant studies is illustrated in Figure 1. A total of 1172 studies were identified from the initial search, of which 128 duplicates and 94 non–English language articles were removed. Titles and abstracts of the 950 remaining studies were screened in accordance to predefined inclusion criteria, and 938 studies were excluded. There was no additional study identified from a citation search, and 12 full-text articles were assessed for eligibility. Eventually, 6 studies were included,^2-4,8,11,14^ while 4 biomechanical studies and 2 meta-analyses were removed. Two studies were based on the same patient cohort, but both were included given that they reported different parameters, which were all of critical clinical relevance.

### Methodology Assessment

The MINORS score for studies ranged from 21 to 22, with a mean value of 21.6. Individual scores for each criterion are detailed in Appendix 1 (available in the online version of this article).

### Demographics

A total of 334 patients undergoing MAT were included in this study. All studies allocated patients to 1 of 2 groups: a suture fixation group and a bony fixation group. Of the 334 patients, 184 underwent MAT with suture fixation and 150 underwent MAT with bony fixation. The mean age in the suture fixation group was 33 years and that in the bony fixation group was 32 years. The mean follow-up of all studies ranged from 2.1 years^11^ to 5.3 years,^8^ and the minimum follow-up ranged from 1.0 years^4,11^ to 2.5 years.^14^ Further study details are displayed in Table 1.

**Table 1:** Baseline Descriptive Characteristics and Details of Randomized Controlled Trials^*a*^

		No. of Patients									
		Suture Fixation			Bone Fixation			Mean Age, y		Follow-up, y	
First Author	Comparative Study	Male	Female	Total	Male	Female	Total	SF	BF	Mean	Minimum
Abat (2013)^2^	Prospective	9	24	33	22	33	55	49	36	5.0	2.5
Bhattacharyya (2023)^4^	Retrospective	50	31	81	20	11	31	27	27	—	1.0
Faivre (2014)^8^	Retrospective	4	7	11	2	10	12	26.7	28	5.3	1.8
Koh (2018)^11^	Case-control	—	—	45	—	—	37	31.2	32	3.0	2.8
Masferrer-Pino (2018)^14^	Prospective	10	4	14	9	6	15	41	35	2.1	1.0

a Dashes indicate data not available.

### Clinical Outcomes

A range of clinical outcomes was measured. Four studies reported on the Lysholm score.^2,4,11,14^ Abat et al^2^ indicated a better Lysholm score in the bony fixation group as compared with the suture fixation group (91.2 vs 88.6; *P* = .001). The same trend was observed by Masferrer-Pino et al^14^ who noted a better Lysholm score in the bony fixation group (94.33 vs 91.43 [*P* = .001]). Bhattacharyya et al^4^ showed the same Lysholm score for suture fixation and bony fixation (85; *P* = .001). The Tegner score was reported by 4 studies.^2,4,11,14^ No significant difference was found in 3 studies, depicting equal values for the bony fixation group and the suture fixation group.^4,11,14^ However, Koh et al^11^ showed a slightly better Tegner score in the suture fixation group (2 vs 1; *P* = .001). In terms of the International Knee Documentation Committee score, this clinical outcome was reported by 3 studies.^4,8,11^ Faivre et al^8^ and Koh et al^11^ noted better outcomes in the suture-only group (69.5 vs 47.1 [*P* = .001] and 74.2 vs 72.9 [*P* = .001], respectively). In contrast, Bhattacharyya et al indicated better outcomes in the bony fixation group (36 vs 32; *P* = .001). Last, 3 studies reported results for the KOOS for symptoms.^4,8,14^ Faivre et al showed a better KOOS outcome in the suture fixation group as compared with the bony fixation group (80.4 vs 50; *P* = .001), and the same trend was observed by Bhattacharyya et al (25 vs 21; *P* = .001). Masferrer-Pino et al^14^ also displayed this trend (92.01 vs 90.88; *P* = .01) (Table 2).

**Table 2:** Patient-Reported Outcome Measures, Mean Outcomes at the Final Follow-up^*a*^

	Lysholm Score		Tegner Score		IKDC Score		KOOS Symptoms	
First Author	SF	BF	SF	BF	SF	BF	SF	BF
Abat (2013)^2^	88	91	3	3	—	—	—	—
Bhattacharyya (2023)^4^	85	85	2	2	32	36	25	21
Faivre (2014)^8^	—	—	—	—	70	47	80	50
Koh (2018)^11^	76	76	1	2	74	73	—	—
Masferrer-Pino (2018)^14^	91	94	7	7	—	—	92	91

a Dashes indicate data not available. BF, bone fixation; IKDC, International Knee Documentation Committee; KOOS, Knee injury and Osteoarthritis Outcome Score; SF, suture fixation.

### Complications

**Figure 2.**
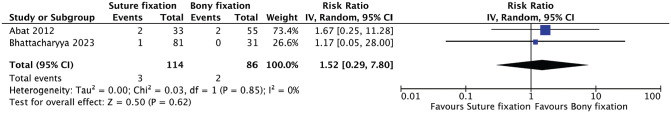
Meta-analysis of infection rates.

**Figure 3.**
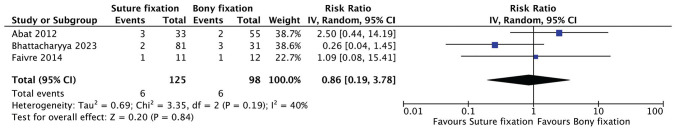
Meta-analysis of graft failure resulting in meniscectomy/graft removal.

**Figure 4.**
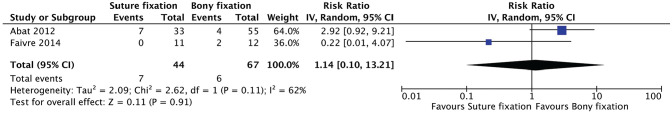
Meta-analysis of graft tears.

**Figure 5.**
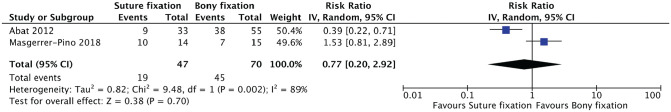
Meta-analysis of minor graft extrusion (≤3 mm).

**Figure 6.**
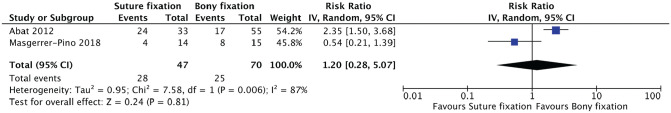
Meta-analysis of major graft extrusion (>3 mm).

Quantitative analysis showed no statistically significant difference in the complications reported between groups. This included risk of infection (relative risk [RR], 1.52; 95% CI, 0.29-7.80; *P* = .62); graft failure (RR, 0.86; 95% CI, 0.19-3.78; *P* = .84); graft tear (RR, 1.14; 95% CI, 0.10-13.21; *P* = .91); minor extrusion, defined as extrusion of graft ≤3 mm (RR, 0.77; 95% CI, 0.20-2.92; *P* = .70); and major extrusion, defined as extrusion of graft >3 mm (RR, 1.20; 95% CI, 0.28-5.07; *P* = .81) (Figures 2-6).

## Discussion

This meta-analysis demonstrated no statistically significant differences in clinical outcomes and complications when comparing suture and bony fixation in MAT. This is an early meta-analysis of case-control studies to compare suture and bony fixation in MAT. The relatively low number of studies available is likely due to MAT not being commonly performed and reserved as a last line of treatment with strict inclusion criteria. Hence, there is a paucity of literature on this topic.

The strength of this analysis is the inclusion of case-control studies to compare 2 fixation methods and their respective outcomes. This strict criterion enhances the quality and reliability of evidence in the efficacy and safety of suture and bony fixation in MAT. It is important to note that the majority of the bony fixation group had lateral MATs, whereas Abat et al^1^ were the only authors who grouped medial and lateral bony fixation. Another strength of this study is the analysis of important outcome variables, such as graft extrusion, graft failure (resulting in meniscectomy/graft removal), and graft tears, which are contraindicative markers of a successful MAT. Reoperation on grafts imposes significant financial, emotional, and social ramifications for patients and the health care system.^19^ Hence, this comprehensive meta-analysis is important for a holistic evaluation of fixation techniques in MAT.

Generally, it is preconceived that bony fixation holds precedence over suture fixation in MAT owing to bone-to-bone healing, resulting in stronger and more rapid healing, fewer root tears, and less extrusion.^5^ Bony fixation is also thought to more closely mimic the contact surface area and contact pressures of the native intact meniscus during the gait cycle.^20^ On the contrary, suture fixation is often regarded as being less dependable because of the potential for the graft and bone to not fully integrate, which may result in a comparatively extended healing process.^20^ Having longer recovery periods can be limiting for patients because of the adjustments that they must make, such as ensuring that the knee remains nonweightbearing and has a restricted range of motion. Therefore, bony fixation is generally more widely favored given the shorter recovery and healing time as compared with suture fixation. Our results have shown no statistically significant difference and hence have disproved this perception.

Graft extrusion after MAT is an adverse outcome that may occur for a myriad of reasons, some of which can be minimized through surgical techniques.^12^ A key reason for graft extrusion is the surgical inaccuracies in identifying the anterior and posterior horn to which the graft is fixated. This may occur because of the loss of normal joint geometry seen in many patients undergoing MAT. Other reasons include the potential size mismatch of the allograft, a reduction in axial trough angle, overtensioning of the suture during surgery, and varied anatomy from patient to patient, which makes it difficult to determine where to place the meniscus during the operation.^6^ Hence, a reliable and solid fixation method is needed in MAT to reduce graft extrusion rates and thereby maximize outcomes.

There are several limitations in this study. Two key outcomes of MAT surgery include graft longevity and associated risk of long-term osteoarthritis progression. Unfortunately, the likelihood of developing osteoarthritis was not an outcome analyzed in this review, and this was mostly due to the short minimum follow-up period (2 years). A longer follow-up period spanning 10 to 20 years will allow for more accurate analysis of the association between each of the 2 fixation methods and the risk of developing osteoarthritis. Moreover, a longer follow-up period will allow for better analysis of which fixation techniques lead to greater risk of minor and major graft extrusion, which, again, is an important clinical outcome as this may dictate rates of reoperation, graft failure, and graft rupture. Ultimately, looking at these outcomes will provide insights into the longevity of each meniscal graft. Last, it is worth recognizing from Figures 2 and 4 to 6 that only 2 studies were included in each of these clinical outcomes. This can be attributed to the limited research currently available in the literature on the topic of MAT fixation methods. Similarly, there is relatively high heterogeneity in clinical outcomes, mainly major and minor graft extrusion, as shown in Figures 5 and 6 (*I*^2^ > 75%). The high heterogeneity observed in these clinical outcomes presents a limitation in our analysis, potentially stemming from variations in study populations, intervention methods, or outcome measurements across the studies. This variability challenges the generalizability of our findings and underscores the need for cautious interpretation of these results.

Patient-reported outcome measures were not able to be analyzed quantitatively given the heterogeneity of raw data collection. A unified and more specific patient-reported outcome measure to assess knee function after MAT may need to be developed in the future. However, various other parameters, such as the Western Ontario and McMaster Universities Osteoarthritis Index, Lequesne Functional Severity Index, and Arthritis Impact Measurement Scales, could provide valuable clinical information that would help determine the difference in clinical outcomes of suture and bony fixation.^10^

This analysis did not account for differences in clinical outcomes arising from variances in surgical techniques within the suture or bony fixation group itself. It is important to note that surgical techniques may vary from one surgeon to another and hence be a confounding factor. Furthermore, having statistical analysis of complications may not fully reflect the true success of the fixation method in MAT. It is important to consider that the success of MAT also pertains to clinical outcomes and being able to meet the expectations and demands of the patient population. Given that expectations may vary from patient to patient, drawing a firm conclusion of a superior fixation method for MAT based on complications is premature and unwise. Additionally, Faivre et al^8^ used a fully open technique for the lateral meniscus during transplantation, and this technique was different to the techniques described in other studies, thereby potentially explaining the relatively higher rate of graft tears observed in this population.

Magnetic resonance imaging (MRI) is the most reliable imaging modality to assess meniscal graft after MAT. Damasena et al^6^ proposed the MRI Appearance in Meniscal Transplant Score system to provide an objective method of evaluating the meniscal graft by MRI appearance. Unfortunately, not all studies used MRI to evaluate the transplanted meniscus, and none of the studies used an objective tool such as the system by Damasena et al to report the state of their graft. Hence, it was difficult to assess for interobserver reliability and external validity of results from individual studies without the use of an objective benchmark and definition.

## Conclusion

No statistically significant differences in functional and clinical outcomes were noted between bony fixation and suture fixation after MAT. However, the short- to medium-term follow-up period is a major limitation to this study. Longer follow-up studies are required to assess the longevity of the meniscal graft and the inherent risk of osteoarthritis progression associated with suture and bony fixation.

## Supplemental Material

sj-pdf-1-ajs-10.1177_03635465251319540 – Supplemental material for Comparison of Suture Versus Bony Fixation in Meniscal Allograft Transplantation Outcomes: A Meta-analysis Supplemental material, sj-pdf-1-ajs-10.1177_03635465251319540 for Comparison of Suture Versus Bony Fixation in Meniscal Allograft Transplantation Outcomes: A Meta-analysis by Rahul Kakria, James Randolph Onggo and Iswadi Damasena in The American Journal of Sports Medicine
