# Supplementary material for: Comparison of Suture Versus Bony Fixation in Meniscal Allograft Transplantation Outcomes: A Meta-analysis
Source: Am J Sports Med. 2025 Mar 3;53(12):3003–9. doi: 10.1177/03635465251319540 (PMC12489164; doi:10.1177/03635465251319540)
Supplement: sj-pdf-1-ajs-10.1177_03635465251319540 – Supplemental material for Comparison of Suture Versus Bony Fixation in Meniscal Allograft Transplantation Outcomes: A Meta-analysis [file sj-pdf-1-ajs-10.1177_03635465251319540.pdf]

Comparison of suture versus bony fixation in meniscal allograft transplantation (MAT) outcomes: A meta-analysis

Appendix 1. MINORS scoring for methodology assessment of included studies

Articles	Year	Clearly stated aim	Inclusion of consecutive patients	Prospective collection of data	Endpoints appropriate to study aims	Unbiased assessment of endpoints	Follow-up period appropriate	Loss to follow-up less than 5%	Prospective calculation of study size	Adequate control group	Contemporary groups	Baseline equivalence of groups	Adequate statistical analyses	Total
**<u>Prospective Studies</u>**														
Abat et al.	2012	2	2	1	2	2	1	2	2	1	2	2	2	21
Masferrer-Pino et al.	2018	2	1	2	2	2	2	2	2	2	2	1	2	22
**<u>Retrospective Studies</u>**														
Bhattacharyya et al.	2022	2	2	2	2	2	1	2	2	2	2	1	2	22
Faivre et al.	2014	2	2	1	2	2	2	2	2	1	2	0	2	21
Koh et al.	2018	2	2	1	2	2	2	2	2	1	2	1	2	22

2: adequately reported; 1: inadequately reported; 0: not reported
